# Supplementary material for: Asymmetric Synthesis of Quaternary Hydantoins via a Palladium-Catalyzed Aza-Heck Cyclization
Source: J Am Chem Soc. 2025 Nov 14;147(49):44692–8. doi: 10.1021/jacs.5c16022 (PMC12703750; doi:10.1021/jacs.5c16022)
Supplement: Supplementary file 2 [file ja5c16022_si_002.zip › All NMR FID Files/21/21_ProtonNMR/1/pdata/1/email_EM05-228-1-1H_1_1.pdf]

qPROTON CDCl3 /opt/nmrdata emeck 18

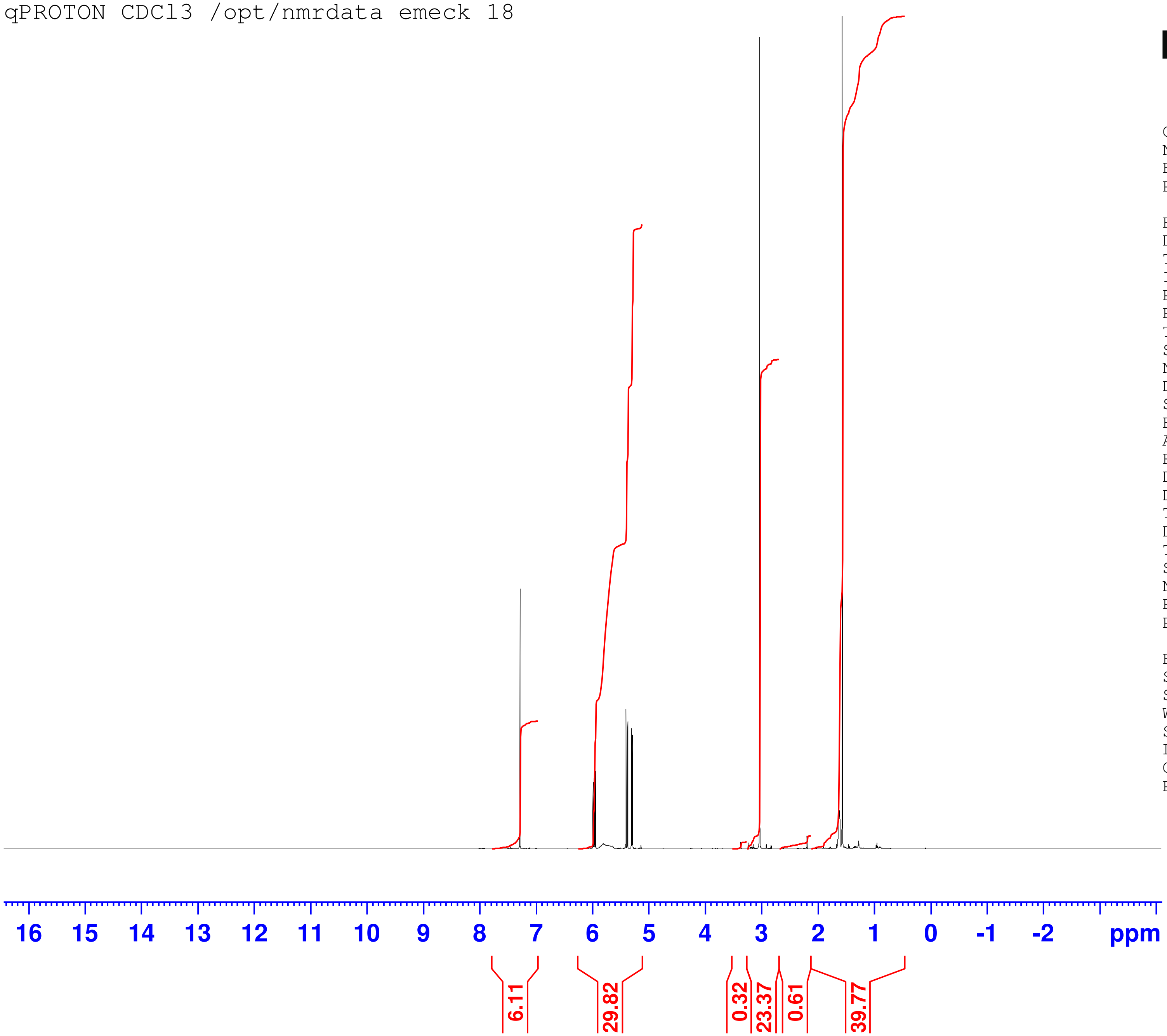

BRUKER

Current Data Parameters

NAME	EM05-228-1-1H
EXPNO	1
PROCNO	1

F2 - Acquisition Parameters

Date_	20240904
Time	11.51 h
INSTRUM	spect
PROBHD	Z168773_0053 (
PULPROG	zg
TD	65536
SOLVENT	CDCl3
NS	16
DS	2
SWH	12335.526 Hz
FIDRES	0.376450 Hz
AQ	2.6563926 sec
RG	64
DW	40.533 usec
DE	18.00 usec
TE	298.0 K
D1	10.00000000 sec
TD0	1
SFO1	600.3237072 MHz
NUC1	1H
P1	12.00 usec
PLW1	21.85499954 W

F2 - Processing parameters

SI	65536
SF	600.3200000 MHz
WDW	EM
SSB	0
LB	0.30 Hz
GB	0
PC	1.00
